# Photopharmacology of Proteolysis-Targeting Chimeras: A New Frontier for Drug Discovery

**DOI:** 10.3389/fchem.2021.639176

**Published:** 2021-03-10

**Authors:** Shenxin Zeng, Hongjie Zhang, Zhengrong Shen, Wenhai Huang

**Affiliations:** ^1^ School of Pharmacy, Hangzhou Medical College, Hangzhou, China; ^2^ Key Laboratory of Neuropsychiatric Drug Research of Zhejiang Province, Institute of Materia Medica, Hangzhou Medical College, Hangzhou, China

**Keywords:** photopharmacology, light, photoPROTACs, drug discovery, proteolysis-targeting chimera

## Abstract

Photopharmacology is an emerging field that uses light to precisely control drug activity. This strategy promises to improve drug specificity for reducing off-target effects. Proteolysis-targeting chimeras (PROTACs) are an advanced technology engineered to degrade pathogenic proteins through the ubiquitin-proteasome system for disease treatment. This approach has the potential to target the undruggable proteome via event-driven pharmacology. Recently, the combination strategy of photopharmacology and PROTACs has gained tremendous momentum for its use in the discovery and development of new therapies. This review systematically focuses on PROTAC-based photopharmacology. Herein, we provide an overview of the new and vibrant research on photoPROTACs, discuss the advantages and disadvantages of this approach as a biological tool, and outline the challenges it faces in a clinical setting.

## Introduction

Light is fast, remote, and easily controllable and can influence the bioactivity of chemical molecules by changing their physicochemical, pharmacodynamic (PD), and pharmacokinetic (PK) properties (Hull et al., 2018). The combination of photochemistry and pharmacology is termed “photopharmacology” (Lerch et al., 2016), which is a nascent field that uses light to control biological systems with high spatial and temporal resolution (Gandioso et al., 2016; Lerch et al., 2016; Li et al., 2019). Photopharmacology is aimed at the improvement of localized specificity and the reduction of off-target-related side-effects of pharmacological agents (Velema et al., 2014). One of the uses of photopharmacology is “color dosing,” in which a specific wavelength (color) of incident light is used to control the concentration of active molecules (Fuchter, 2020; Meijer et al., 2020). Because of the unique feature of color dosing in photopharmacology, photopharmacology has been widely used in chemical biology, psychiatric research, and disease treatment, such as vision restoration (Bamberg et al., 2018; Hull et al., 2019; Silva et al., 2019).

Currently, several strategies are used to disrupt the function of intracellular proteins, including DNA modification and RNA interference, which affect proteins at the level of the genome and mRNA, respectively (Clift et al., 2017). Unfortunately, neither DNA modification nor RNA interference can regulate proteins reversibly and rapidly, thereby restricting their use in clinical practice (Wang Z. W. et al., 2020). Recently, a proteolysis-targeting chimera (PROTAC) strategy has emerged, with a currently unsurpassed ability to reversibly and rapidly induce the degradation of a protein of interest (POI) through the ubiquitin-proteasome system (Liu et al., 2020a). A PROTAC molecule is composed of three essential components as follows: a POI binder that specifically binds the target protein, a ligand of the E3 ligase that recruits the corresponding E3 ubiquitin ligase, and a linkage vector that ensures the correct orientation of and the distance between the two ligands (Toure and Crews, 2016; Lai and Crews, 2017). The unique chemical structure of PROTAC dictates its biological activity. Regarding the mode of action (MOA), a PROTAC molecule elicits activity in an “event-driven” manner, and the requirements for binding affinity and binding sites are not strict (Wang Y. et al., 2020). Therefore, PROTAC technology is considered a powerful strategy for converting intractable targets into druggable ones. Indeed, it has already been demonstrated to induce the degradation of K-Ras (Bond et al., 2020; Zeng et al., 2020) and signal transducer and activator of transcription 3 (STAT3) (Zhou et al., 2019). Additionally, the protein-protein interaction (PPI) between the POI and E3 ubiquitin ligase improves selectivity to the target protein, and this has been shown by the depletion of cyclin-dependent kinase 6 (CDK6) (Brand et al., 2019), bromodomain-containing protein 9 (BRD9) (Zoppi et al., 2019), histone deacetylase 6 (HDAC6) (Wu et al., 2019), B-cell lymphoma-extra large (BCL-X_L_) (Khan et al., 2019; He et al., 2020a; He et al., 2020b; Khan et al., 2020; Zhang et al., 2020), and Wee1 (Li et al., 2020). Furthermore, PROTAC technology has shown promising results in overcoming acquired drug resistance as shown by targeting breakpoint cluster region protein- (BCR-) ABL to treat imatinib-resistant chronic myelomonocytic leukemia (Shimokawa et al., 2017), androgen receptor to treat enzalutamide-resistant prostate cancer (Shibata et al., 2018), and Bruton’s tyrosine kinase to treat ibrutinib-resistant lymphoma (Tinworth et al., 2019).

In 2001, Crews conducted pioneering research on PROTACs (Sakamoto et al., 2001). This first generation was peptide-based, and its poor cell permeability and stability limited its application to translational medicine. After decades of development, the second generation of PROTACs emerged (Testa et al., 2018). PROTAC technology has since progressed tremendously. At least 60 proteins have been successfully degraded by a corresponding PROTAC molecule (Zeng et al., 2020), and, in 2019, two oral PROTACs, ARV-471 (NCT04072952) and ARV-110 (NCT03888612), have advanced into clinical trials for the treatment of metastatic breast cancer and prostate cancer, respectively (Mullard, 2019; Wang Y. et al., 2020). These achievements in PROTAC development have encouraged further research in both academia and the pharmaceutical industry (Sun et al., 2019). Owing to these efforts, the knowledge database on PROTACs has grown tremendously over the last decade, and several reviews on the progress of PROTACs development are available. In our previous study, we have systematically reviewed the opportunities and challenges of the PROTAC technology (Zeng et al., 2021). A recent study published by Bayer presented a comprehensive overview of PROTAC-induced target protein degradation from the perspective of medicinal chemistry (Luh et al., 2020). Various oncogenic proteins have also been successfully targeted by PROTACs and the potential ability to treat various hematologic malignancies was summarized by He et al. in 2020 (He et al., 2020).

Despite these unique features of PROTACs (Burslem et al., 2018), the PROTACs have no capacity for spatiotemporal control, which would dramatically reduce the incidence of off-target effects (Churcher, 2018), given that most pharmacological protein targets are widely expressed in both healthy and diseased tissues or are overexpressed in localized diseased tissues and diffuse to healthy tissues. Systemic administration and the catalytic nature of these PROTACs can affect nontarget tissues and result in adverse effects, which limit their application in clinical practice. For example, a recent study has shown that mice treated with ARV-711, BRD4 degrader, exhibited spine hunching, lethargy, and decreased mobility (Raina et al., 2016). Therefore, the development of a new therapeutic method that allows for remote activation at a specific site and a specific time, irrespective of target distribution, is urgently required.

**FIGURE 1:**
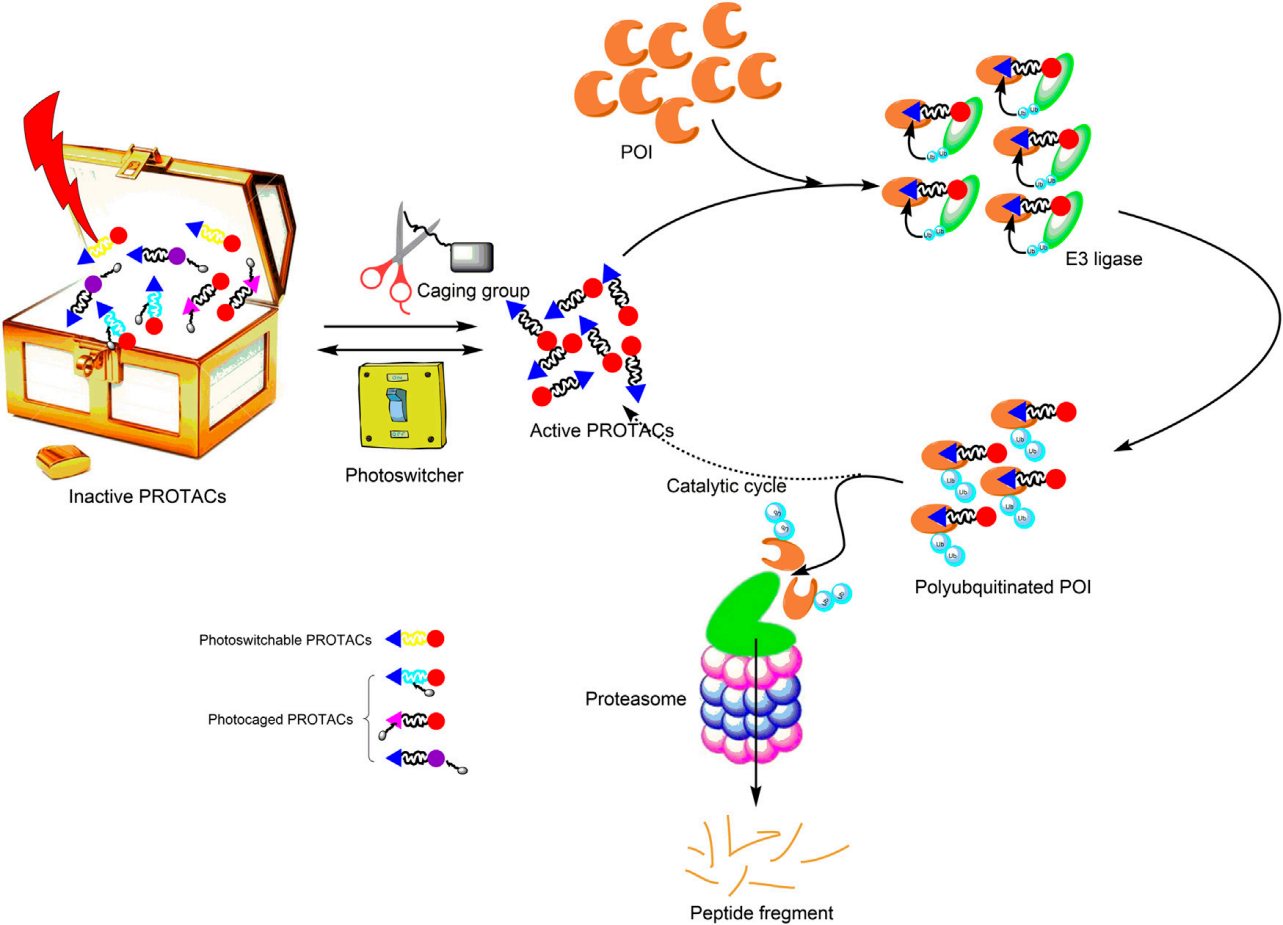
Principle of photoPROTACs. Upon irradiation with an appropriate wavelength of light, photoPROTACs are activated. (Photoswitchable PROTACs undergo conformation change into active PROTACs, and photocaged PROTACs are induced to dissociate from the caging group to expose active functional groups.) Subsequently, the activated PROTACs bind the POI and recruit relevant E3 ligase for ubiquitination. Ultimately, polyubiquitinated POIs are degraded via the proteasome pathway. Active PROTACs separate from the polyubiquitinated POIs and participate in the subsequent catalytic cycle. Gray rectangle represents the caging group; yellow linker signifies the photoswitcher element; The black linker conjugates the cyan triangle with the red circle and presents the active PROTAC. POI, protein of interest; PROTAC, proteolysis-targeting chimera.

Recently, the third generation of PROTACs—controllable PROTACs—was proposed (Pfaff et al., 2019; Xue et al., 2019; Kounde et al., 2020; Liu et al., 2020a; Wu and Manna, 2020). The combination of photopharmacology and the PROTAC technology (photoPROTAC) enables the spatiotemporal control of protein degradation, which should reduce toxicity, improve selectivity, establish tissue specificity, and eliminate off-target effects. Therefore, the photoPROTAC technology provides an opportunity for the development of a novel strategy for precision medicine (Figure 1).

The effects of light on PROTACs can be irreversible or reversible, and both effects have been explored (Gautier et al., 2014). Here, we focus on both reversible and irreversible activity of chimeric molecules for targeted degradation with spatial and temporal control. Subsequently, the advantages and disadvantages of the photoPROTAC technology are discussed. Lastly, the challenges involved in the use of this technology in translational medicine are presented.

## Progress of PhotoPROTAC Development

Recently, the development of photoPROTACs has been the focus of several researchers and has been receiving increasing interest (Graupner et al., 2018; Heindl and Wegner, 2020; Pugachev et al., 2020). After retrieval of comprehensive information, we found that photochromic and photocleavable functional groups are most extensively used in photoPROTAC development. Thus, photoPROTACs can be divided into two categories, namely, photoswitchable PROTACs and photocaged PROTACs (Hoorens and Szymanski, 2018; Kounde and Tate, 2020; Nalawansha and Crews, 2020).

### Photoswitchable PROTACs

**FIGURE 2:**
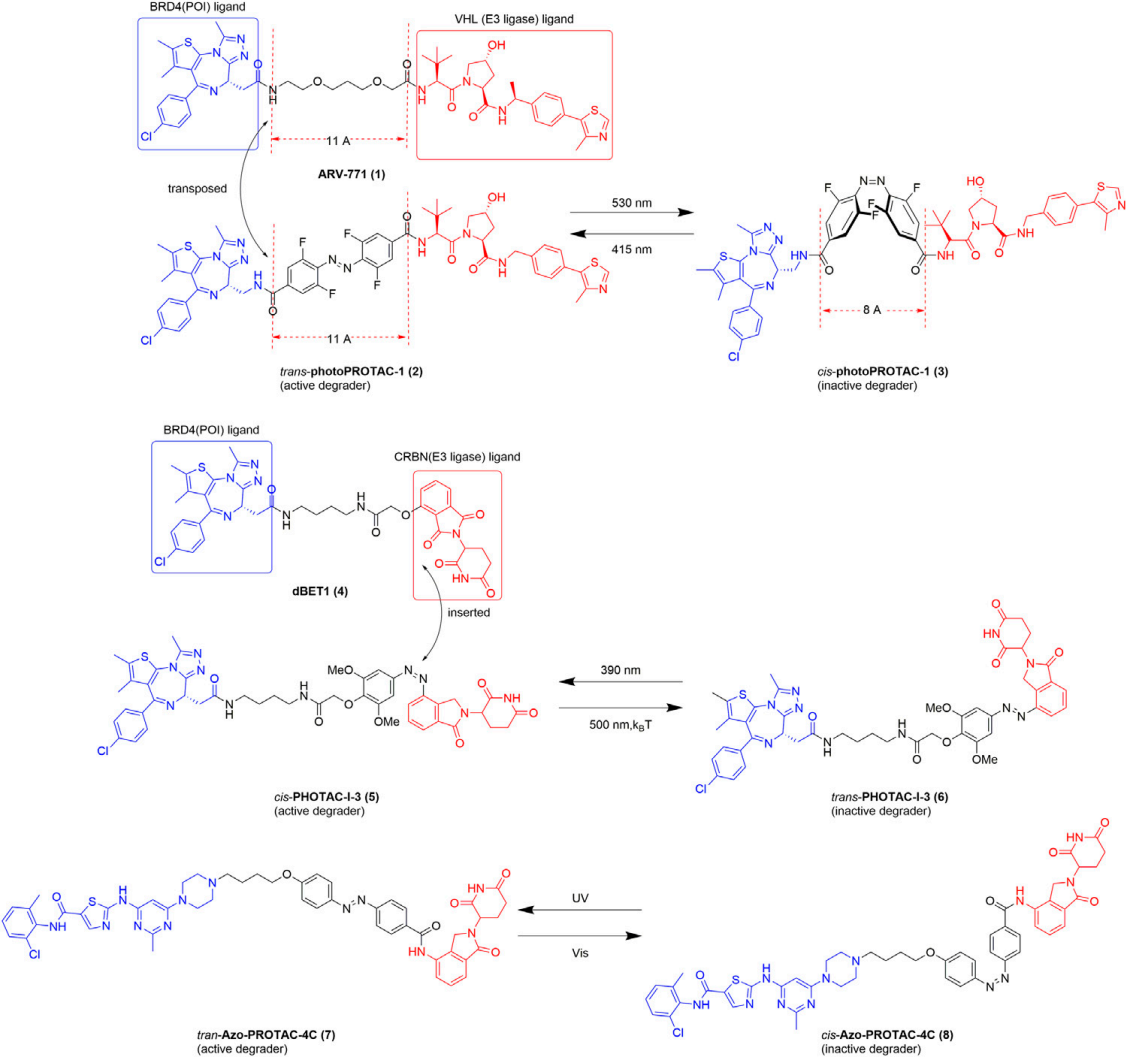
Structures of the representative photoswitchable PROTACs. BRD4, bromodomain-containing protein 4; VHL, von Hippel–Lindau; CRBN, cereblon (Pfaff et al., 2019; Jin et al., 2020; Reynders et al., 2020).

A photoswitchable PROTAC is based on the PROTAC platform and enables the reversible on/off switching of protein degradation (Verma and Manna, 2020; Harris et al., 2018). The first photoswitchable PROTAC was developed by Crews in 2019 (Pfaff et al., 2019). Spatiotemporal control of protein degradation using a PROTAC was achieved after the insertion of an *ortho*-F_4_-azobenzene linker functionality between the BRD4 and von Hippel–Lindau (VHL) ligands (Figure 2). *Trans*-photoPROTAC-1 (2) is an active degrader, whereas its *cis* isomer is inactive because the topological distance between the ligands in the latter is inappropriate for the ubiquitination of the target protein. In contrast, the *azo-trans*-isomer facilitates the formation of a necessary and productive ternary complex. Interestingly, several examples of PROTACs show that the critical difference in linker length between the active and inactive isomers is approximately 3 Å (Buhimschi et al., 2018; Zhou et al., 2018). The difference in topological distance between the *trans*- and *cis*-azobenzenes (3–4 Å) is consistent with those reported previously. Consequently, the linear polyether linker in ARV-771 (1) was replaced with azobenzenes, and the novel structure of the photoPROTAC was developed. Considering potential applications, the lifetime of the photostationary state is an important parameter. With a relatively long-lived photostationary state (∼days), the typical requirement for continued or pulsed irradiation exposure can be circumvented (Pfaff et al., 2019). A previous report shows that *ortho*-tetrafluoroazobenzenes (*o*-F_4_-azobenzenes) have a bistable nature. Thus, these azobenzenes were used to optimize the photoPROTAC design (Bleger et al., 2012). After a series of systematic design changes and optimization steps, a putatively photoswitchable PROTAC was yielded, which can be used as an alternative strategy for constructing bistable photoPROTACs.

With the goal of reversible control of protein degradation, Reynders *et al.* recently achieved a series of photoswitchable PROTACs by introducing an azobenzene functionality to the PROTAC (Reynders et al., 2020). In this system, all synthetic chimeras exhibit little or no degradation activity prior to light exposure but are converted into the active state with the application of blue-violet light (380–440 nm) (Figure 2). With the establishment of a small library of azo-based PROTACs, a representative compound, PHOTAC-I-3, emerged as one of the most potent light-controlled degraders. Interestingly, in contrast to the results of the study by Crews, *cis-*PHOTAC-I-3 (5) was found to be an active degrader. To explain this difference, we should be aware of the importance of *o*-F_4_ and that some groups on the *ortho*-position of azobenzenes can alter photophysicochemical properties and bioactivity of PROTACs. Importantly, at the maximum degradation wavelength (390 nm) >90% exists as the *cis isomer*, whereas at wavelengths >450 nm, >70% exists as the *trans isomer*. Notably, *cis-*PHOTAC-I-3 (5) has a desirable half-life of 8.8 h at 37°C in dimethyl sulfoxide and has the advantage of fatigue resistance. Furthermore, western blot analysis showed that PHOTAC-I-3 (5, 6) degrades BRD proteins only at a wavelength of 390 nm. When treated with 10 µM PHOTAC-I-3 (5, 6) at 390 nm irradiation, a “hook effect” is observed, which is consistent with previous reports. Most importantly, the topological distance between dBET1 (4) and *cis*-PHOTAC-I-3 (5) is unequal, which is slightly different from that observed by Crews. We speculate that one of the main reasons the isomers have similar degradation activity despite the difference in topological distance is that the *cis* isomer of the azo-based linker can alter the orientation required for ubiquitination (Wu and Manna, 2020).

Recently, a library of novel Azo-PROTAC structures was established through the incorporation of azobenzene moieties into the linker (Jin et al., 2020). Azo-PROTAC-4C (7, 8), a representative compound, exhibits reversible and controllable degradation in intact cells in response to light irradiation (Figure 2). A BCR-ABL fusion protein degradation assay showed that the *trans*- and *cis*-configurations of Azo-PROTAC-4C have significantly different abilities to degrade the POI. Specifically, treatment with 100 nM of the *trans* isomer results in the degradation of BCR-ABL, whereas no degradation of the POI occurred using the *cis* isomer in similar experimental conditions. However, only UV, which has poor cell penetrability and cell-damaging properties, can switch the configuration of Azo-PROTAC-4C, thereby impeding its future development and use. However, this is still an expanding area of study, and further research is underway to overcome the current limitations of the system.

In conclusion, photoswitchable PROTACs are inactive under certain conditions and become active under appropriate wavelengths of light. This unique trifunctional chimeric molecule is capable of controlling POI degradation with spatiotemporal precision. Interestingly, it can be switched on/off thousands of times with fatigue resistance. PROTACs have been the focus of research in the past 2 years, and we firmly believe that photoswitchable PROTACs have the potential to be utilized as both chemical tools and therapeutic agents.

### Photocaged PROTACs

Photocaged PROTACs are complementary to photoPROTACs. They bear a photolabile protecting group to prevent interactions with either the POI or the E3 ligase, which is irreversibly released upon irradiation with certain wavelengths of light irradiation. For spatial and temporal control over POI degradation, many scientific studies on photocaged PROTACs have been reported (Xue et al., 2019; Vorobev and Moskalensky, 2020).

Several approaches are conceivable for the installation of a caging group on PROTACs: 1) they could be added as a part of the E3 ligase ligand to reduce the affinity of the chimera to the E3 ligase; 2) they could be installed on the tether between warheads to affect the PPI between the POI and the E3 ligase; 3) they could be added to the POI ligand to block interaction with the POI.

**FIGURE 3:**
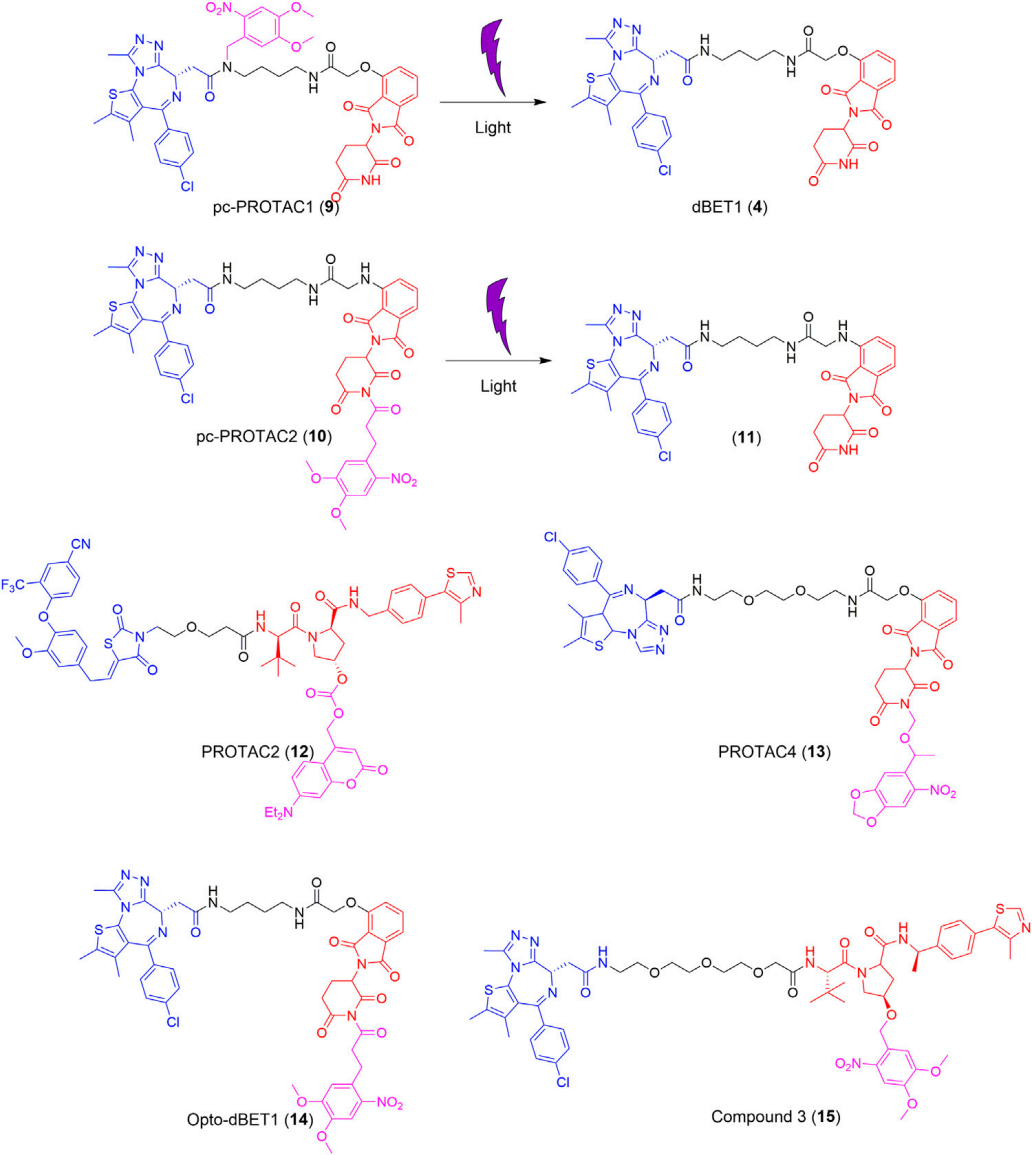
Structures of the representative photocaged PROTACs (Xue et al., 2019; Kounde et al., 2020; Liu et al., 2020b; Naro et al., 2020).

dBET1 (4), developed in 2015, is typically used as a lead compound for structure optimization or as a positive control for BRD4 degradation (Winter et al., 2015). Based on dBET1 (4), a bulky 4,5-dimethoxy-2-nitrobenzyl (DMNB) group was installed on the amide nitrogen of the JQ1 moiety and the imide nitrogen of the thalidomide moiety to synthesize pc-PROTAC1 (9) and pc-PROTAC2 (10), respectively (Figure 3) (Xue et al., 2019). Upon irradiation at 365 nm, 50% of pc-PROTAC1 disintegrates into the desired product, dBET1 (4), whereas no dBET1 (4) is released in the dark. However, under similar conditions, no dBET1 (4) is detected when pc-PROTAC2 (10) is irradiated with light. After the incorporation of the DMNB group in JQ1, the binding affinity of pc-PROTAC1 (9) to BRD4 decreases more than 100-fold compared to that of pc-PROTAC2 (10). A degradation experiment shows that pc-PROTAC1 (9) degrades BRD4 in a light-dependent fashion. The degradation of BRD4 is obviously observed after light irradiation at 365 nm for only 0.3 min and is completely degraded after 3 min. The evaluation of zebrafish embryos *in vivo* showed that BRD4 protein is significantly degraded in embryos treated with pc-PROTAC1 (9) (50 or 100 μM) with light irradiation at 365 nm, which confirms the light-induced degrading activity of pc-PROTAC1 (9) in zebrafish. These findings, therefore, provide an alternative strategy for the spatial and temporal control of protein function.

Recently, two different light-cleavable caging groups were installed on VHL and cereblon (CRBN) E3 ubiquitin ligase ligands (Naro et al., 2020). Diethylamino coumarin (DEACM) was installed on the hydroxy of the VHL ligand, which was converted into PROTAC 2 (12), while, 6-nitropiperonyloxymethyl (NPOM) was installed on the imide nitrogen of the CRBN ligand, which yielded PROTAC 4 (13) (Figure 3). The mechanism of the photocleavage of DEACM shows that it can be photodecomposed with ≤405 nm light irradiation and releases an acidic functional group at a pK_a_ lower than 5. The photocaging group, NPOM, shows efficient photolysis in the presence of 365 nm light and has been used in numerous biological studies. Overall, the degradation of PROTACs can be completely suppressed by introducing an appropriate caging group and this effect can be reversed with irradiation at a suitable wavelength.

More recently, a photocaged PROTAC was developed (termed opto-dBET1, 14) (Figure 3), which has a slightly different structure from that of pc-PROTAC1 (9) (Liu et al., 2020b). The main difference between opto-dBET1 (14) and pc-PROTAC1 (9) is the position of the DMNB group. In the development of opto-dBET1 (14), the caging group, DMNB, was introduced to the NH group of the pomalidomide moiety, which blocks the formation of a hydrogen bond between the glutarimide NH of pomalidomide and the backbone carbonyl of His^380^ in CRBN. Biochemical and biological validation and characterization showed that irradiation with light controls the uncaging of opto-dBET1 (14) to regulate the degradation of BRDs in a timely and dose-dependent manner.

Similarly, the DMNB caging group was introduced to the VHL ligand and compound 3 (15) was generated (Figure 3) (Kounde et al., 2020). As anticipated, compound 3 (15) results in the dose-dependent degradation of BRD4 only upon irradiation. Importantly, compound 3 (15) exhibits good stability (5 days) in both solution and the cellular environment in the absence of light.

## Potential Challenges for PhotoPROTACs

The development of an effective photoPROTAC for clinical application should fulfill multiple criteria, including the need for a biologically inert parent drug, clean photoreaction, byproducts, and parent compounds with low toxicity, high quantum yield, and high molar attenuation coefficient. Additionally, the parent drug should be stable and no free radicals should be formed, and the photoPROTAC should exhibit thermal stability. Secondly, the PROTAC should facilitate photocaging and an appropriate wavelength of light should be selected for irradiation. Lastly, a specific photoreaction should be selected and the photoreaction should be rapid to avoid overirradiation and unwanted reactions. Therefore, the development of photoPROTACs is a challenging process during the drug discovery pipeline (Fuchter, 2020). However, the resolution of these problems will provide an unprecedented opportunity in the field of precision medicine.

### Extension of Photosensitive Elements

**FIGURE 4:**
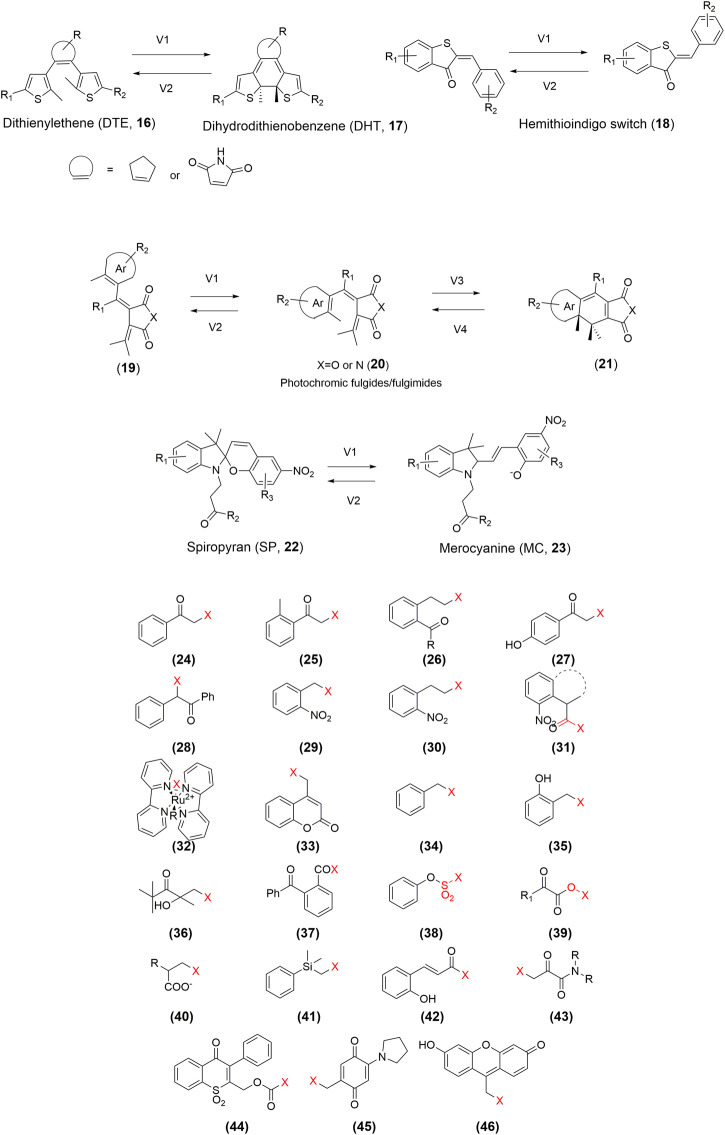
Structures of the representative photoPROTAC elements.

Despite the progress in the development of photoPROTACs, the photoactive moiety lacks diversity. Developing an effective photoPROTAC largely depends on the successful identification of the pivotal photoactive functional groups. Currently, the design of photoswitchable PROTACs mainly focuses on the azo-based switcher, whereas that of photocaged PROTACs is mostly based on the installation of the DMNB, DEACM, or NPOM groups. So far, several photosensitive molecules have already been studied *in vivo*. Expanding the photoactive moiety can facilitate the development of improved photoPROTACs. For instance, a bistable diaryl-ethene photoswitch (Falenczyk et al., 2014; Presa et al., 2015), a hemithioindigo switch (Lachmann et al., 2017), photochromic fulgides/fulgimides (Lachmann et al., 2017), and photochromic spiropyran (Velema et al., 2015) have been extensively used in photopharmacology (Figure 4). Furthermore, as shown in Figure 4, multiple photocaging groups have already been developed (Klán et al., 2013). However, whether these chemical toolboxes are suitable for the development of desirable photoPROTACs requires validation. Additionally, more novel photoswitchable and photocaging functional groups with low toxicity and high potency need to be developed urgently.

### Innovation of Light Delivery System

PhotoPROTACs induce POI degradation in a light-dependent manner; therefore, the delivery of light is essential. However, there is a barrier to this technique. For instance, low energy of UV-Vis light is susceptible to absorption by endogenous substances, scattered in tissue, and blocked owing to the depth of tissue (Gienger et al., 2020). Furthermore, high-intensity light, especially UV, triggers photodamage in cells and accelerates gene mutation, which may have significant impacts on health. Thus, how to deliver photons to the target tissues is a persistent issue. Fortunately, photodynamic therapy (PDT), which has been developed over several decades to overcome the problems related to light delivery (Silva et al., 2019), could provide solutions. The many successful clinical applications of PDT have demonstrated the feasibility of this photopharmacological approach. Moreover, PDT could be further developed along two parallel pathways. First, from an instrumental perspective, PDT could inspire the modification and creation of equipment for clinical use, such as the development and improvement of new light sources, including lasers and LEDs. Moreover, a combination of current technologies, such as light diffusers, fiberoptic devices, and computer-aided delivery systems, can also be considered. Second, from a chemistry perspective, medicinal chemists should design PROTAC molecules that can be activated at 650–900 nm because this wavelength is optimal for deep-tissue penetration and near-infrared wavelengths are known to cause comparatively little photodamage. A chemical structure that enables sufficient red-shift to allow optical irradiation in the near-infrared radiation or infrared radiation window for optimal tissue penetration must also be designed. In line with these requirements, a number of azobenzene-based photopharmacological agents have recently been reported to be switched on/off upon exposure to the desired wavelength.

### Improvement of Physicochemical Properties

Physicochemical properties are important for a potential drug. The key physicochemical features of photoPROTAC include a generally high molecular weight, high polar surface area, and poor chemical stability, solubility, and permeability. Light is everywhere in daily life, and photoswitchable PROTACs and photocaged PROTACs are threatened by light during the manufacture, transportation, and administration processes, which probably affects the absorption, distribution, metabolism, excretion, and toxicity of PROTACs and even the potency of POI degradation (Bonnet, 2018). Moreover, high absorbance and quantum yields and useful thermal relaxation rates are quite important. Consequently, photoPROTACs face more complex physicochemical challenges than traditional small molecules.

### Optimization of Pharmacokinetic (PK)/Pharmacodynamic (PD) Properties

Photochromic ligands, such as azo-based PROTACs, are photosensitive molecules that can change into at least two conformations under light exposure (Vlasceanu et al., 2018). These conformations exhibit different degradation efficacies, different affinities toward their POI, and different PDs. Therefore, the relative thermal stability of the isomers, which may vary greatly in different systems, is extremely important. Indeed, the tautomerism of azo-based PROTACs occurs rapidly in response to exposure to light at the appropriate wavelength, while one isomer in another system can have appreciable thermal stability (Crespi et al., 2019). The stability of the *cis* and *trans* forms are important for medical use. Furthermore, 100% of an isomer can not be converted into the other; therefore, the steady-state relative abundance of *trans* and *cis* isomers is another critical parameter to be considered for azo-based PROTACs.

Poor PK properties are a key cause of drug attrition during clinical development. A potential drug should have a desirable PK profile and favorable metabolic stability in a given environment. Therefore, PK properties have been recognized as a pivotal parameter for drug discovery, including the development of photoPROTACs. Although chemists can easily add functional groups, large molecular size and high molecular weight can seriously limit the oral bioavailability and membrane permeability of a PROTAC. Additionally, as shown in a recent study, traditional methods for PK evaluation cannot be used for photoPROTACs, owing to the substoichiometric MOA of photoPROTACs (Mares et al., 2020).

Upon irradiation, photocaged PROTACs release a casing group that may exert toxicity or other adverse effects; thus, the development of a non-toxic casing group should also be considered in the development of photoPROTAC. In contrast, photoswitchable PROTACs isomerize only between the *trans* and *cis* forms and do not involve the formation of other byproducts. However, azobenzenes include compounds that are carcinogenic to humans, such as methyl yellow a food colorant, and prontosil, a prodrug. Although the reputation of azobenzenes may cause concern, other azobenzenes, such as tartrazine, sunset yellow, and Allura red, are still widely used as food colorants, implying that chemical structure optimization could likely result in the consideration of non-carcinogenic azobenzenes in future studies (Hull et al., 2018).

### The Regular Challenges for PhotoPROTACs

PhotoPROTACs were designed based on regular PROTACs, so some of the conventional challenges to PROTACs also apply to photoPROTACs. The available information on the rational design of PROTACs is sparse. Therefore, expanding the understanding of the rational drug design for PROTACs is important for efficient photoPROTACs development. Fortunately, an online database of PROTACs was recently established by Weng et al., which may promote the rational design of PROTACs (Weng et al., 2021). E3 ligase is quite important for POI degradation; although more than 600 E3 ligases have been reported, less than 1% of them are recruited by small-molecule ligands (Konstantinidou et al., 2019). Unfortunately, most reported PROTACs or photoPROTACs are based on CRBN, VHL, mouse double minute 2 homolog (MDM2), and cellular inhibitor of apoptosis protein 1 (cIAP1). Thus, the main challenge in the design of potent PROTACs with novel structures and good drug-like properties is the expansion of the repertoire of E3 ligase ligands. In addition, the complexity of PROTACs with high molecular weight and instability can impact cellular permeability. Excitingly, with the multidisciplinary involved, sustainable oral active PROTACs were developed.

Research on photoPROTACs is still in a stage of infancy and there is not much available literature about the optimization of the PD/PK profiles. However, recently, Roche and C4 Therapeutics have jointly published an article on the optimization of the drug metabolism and pharmacokinetics of traditional PROTACs (Cantrill et al., 2020). Unfortunately, owing to the unique structure of photoPROTACs, the current knowledge and optimization strategies are not completely applicable to this trifunctional molecule. However, we firmly believe that, in the near future, the challenges to photoPROTAC development will be overcome and will enable the clinical use of photoPROTACs.

## Conclusions and Prospects

PhotoPROTACs are unparalleled in their ability to degrade disease-causing proteins via the ubiquitin-proteasome system with high spatial and temporal resolution. Currently, the most popular methods to generate photoPROTAC molecules are to incorporate a photochromic functional group, such as azobenzenes, that can be activated when exposed to light, or to install a photocleavable group, such as DMNB that is cleaved upon light exposure. With azobenzenes, the reversible reaction is rapid (usually a few seconds), and thousands of conversions occur without fatigue. Regarding photocaged PROTACs, a number of caging groups have been identified and can be used to establish a photocaged PROTAC compound library.

Although remarkable achievements have been made with photoPROTACs, which are promising tools for disease treatment, the challenges to the light delivery system, the physicochemical properties, and the PK/PD properties must be overcome before this technique can be used in clinical practice.

PhotoPROTACs, although not yet in clinical development, have the potential to become a powerful tool therapeutic in precision medicine, owing to their ability to degrade targets in a photocontrolled manner with high spatiotemporal precision. Furthermore, vision restoration has opened a new avenue for other applications of photopharmacology and the first oral active PROTAC, ARV-110, could also pave the way for the development of more PROTACs. Therefore, we are optimistic that the combination of photopharmacology and PROTACs will eventually be used in human precision medicine.
